# Deciphering the Complexity of Smoke Point in Virgin Olive Oils to Develop Simple Predictive Models

**DOI:** 10.3390/foods14234099

**Published:** 2025-11-28

**Authors:** Anna Díez-Betriu, Beatriz Quintanilla-Casas, Josep J. Masdemont, Alba Tres, Stefania Vichi, Francesc Guardiola

**Affiliations:** 1 Departament de Nutrició, Ciències de l’Alimentació i Gastronomia, Campus de l’Alimentació de Torribera, Facultat de Farmàcia i Ciències de l’Alimentació, Universitat de Barcelona, 08921 Santa Coloma de Gramenet, Spain; annadiez@ub.edu (A.D.-B.); beatrizquintanilla@ub.edu (B.Q.-C.); atres@ub.edu (A.T.); fguardiola@ub.edu (F.G.); 2 Institut de Recerca en Nutrició i Seguretat Alimentària (INSA-UB), Universitat de Barcelona (UB), 08921 Santa Coloma de Gramenet, Spain; 3 IEEC & IMTEch Departament de Matemàtiques, Universitat Politènica de Catalunya, 08028 Barcelona, Spain; josep.masdemont@upc.edu

**Keywords:** virgin olive oil composition, smoke point prediction, PLS and Gaussian models

## Abstract

The smoke point marks the onset of thermal degradation in edible oils. Although in this work we validated and improved its determination, it still relies on a subjective visual assessment and remains incompletely understood in relation to oil composition. This limitation reduces its reliability as a criterion for selecting frying oils in both industrial and culinary contexts. This study provides a systematic evaluation of how key chemical attributes of virgin olive oils influence their smoke point and proposes predictive models that could overcome the limitations of direct measurement. Forty-eight virgin olive oils were characterized, and multivariate modeling was applied to identify the most influential predictors. Free fatty acid content was the main determinant of the smoke point, exhibiting a strong inverse relationship, while saturated fatty acids and oxidative stability were shown to increase the smoke point by limiting the formation of volatile lipid oxidation products. Partial least squares models enabled accurate predictions using only routine quality parameters, such as free fatty acid content and saturated fatty acid content. Gaussian process regression further improved predictive performance and achieved high accuracy using free fatty acid content alone or, alternatively, other analytical parameters that are easily and routinely determined in olive oil. These findings offer a potential practical framework for estimating the smoke point without direct testing, with relevant implications for virgin olive oil quality control and the selection of oils for high-temperature applications.

## 1. Introduction

The smoke point is defined as the minimum temperature at which oil releases a thin continuous stream of smoke, a phenomenon directly related to the presence of volatile compounds such as free fatty acids (FFAs) and low-molecular-weight products [1,2]. This parameter is particularly relevant for deep-frying, where high temperatures, moisture from food, and oxygen accelerate hydrolysis, oxidation, and polymerization reactions. These reactions produce volatile compounds that contribute to fried food aroma but may also generate substances with adverse nutritional or health effects [3], including aldehydes such as acrolein and polycyclic aromatic hydrocarbons [4,5,6]. Heating oils to high temperatures has also been linked to the emission of particulate matter, which is considered a contributing factor to numerous health problems [7]. Since the formation of these thermal degradation products increases sharply after the smoke point is reached [4,7], the smoke point is widely used to assess the thermal suitability of frying oils [8]. Values above 200 °C are generally recommended by professional and scientific sources [9,10,11,12], and certain national guidelines specify minimum smoke points for frying applications [13].

During deep-frying, the smoke point generally decreases as degradation products accumulate, particularly free fatty acids resulting from hydrolysis [14,15]. For this reason, several countries recommend smoke point thresholds to guide oil disposal [16,17].

However, the smoke-point parameter presents relevant gaps. First of all, its measurement remains empirical and often poorly reproducible [16]. The AOCS Official Method Cc 9a-48 [1] is the most commonly applied procedure. It relies on visual assessment, which is subjective and can be influenced by experimental conditions. In particular, incandescent lightbulbs, originally specified in the AOCS method, have largely been replaced by LED lighting. This raises the question of whether the color temperature of the light source affects endpoint visualization and, consequently, the accuracy and precision of the smoke-point determination. Moreover, although its widespread use in gastronomy, the smoke point remains insufficiently understood from a scientific perspective, particularly in relation to other quality and compositional parameters. It is well established that the smoke point depends strongly on oil’s FFA content [18,19,20], with a pronounced negative correlation observed [21]. FFAs originate from hydrolysis of triacylglycerols, and their levels are associated with the quality of the raw oil, thereby influencing the smoke point of virgin oils. The oil refining process markedly reduces FFAs and other volatile compounds [22], resulting in refined edible oils having higher and more consistent smoke points than virgin oils. The smoke point is also influenced by fatty acid (FA) composition, particularly chain length: oils rich in shorter-chain FA tend to exhibit lower smoke points than those with longer-chain FA [4]. Furthermore, Yen et al. [23] demonstrated that the smoke point of soybean oil and lard increases upon the addition of synthetic antioxidants. Other researchers have demonstrated the effects of minor seed oil components on the smoke point. Wu et al. [24] reported that the smoke point was negatively correlated with FFAs, phospholipids, and moisture and volatile matter, but positively correlated with the peroxide value. Al-Dabbas et al. [25] observed a decrease in the smoke point of oils as a result of the removal of oxidation products and volatile compounds.

For virgin olive oils, the smoke point is expected to vary according to their FFA content, which can reach up to 2% under EU regulations [26]. Only a limited number of studies have measured the smoke point of virgin olive oils in the context of their thermal performance [20,27,28]; consequently, the relationship between smoke point and other quality and compositional parameters remains unclear. A suitable approach for selecting virgin olive oils for deep-frying and monitoring their degradation is to employ objective and reliable methods based on routinely measured physicochemical and compositional parameters. Using these parameters to model and predict the smoke point allows for minimizing reliance on subjective visual assessment while improving accuracy and reproducibility. To achieve this, it is necessary to establish clear correlations between the smoke point and the compositional parameters that influence it. Predictive models for the smoke point of edible oils have been proposed [24,29], but they rely on a large number of parameters, not always routinely measured, which limits their applicability in routine quality control, or require specific instrumentation. In the case of olive oil, no scientifically validated data or published models are currently available.

To address these gaps, the aim of this study was therefore twofold: (i) to advance understanding of smoke point variation in virgin olive oils by examining its associations with key quality and compositional parameters and (ii) to reassess the current smoke point measurement procedure with the goal of improving reliability and developing predictive models requiring fewer analytical inputs.

## 2. Materials and Methods

### 2.1. Samples

Samples (*n* = 48) of virgin olive oils were obtained during the 2017/18 and 2018/19 campaigns. The sample set included 19 virgin olive oils of the commercial category extra virgin (EVOO) and 29 virgin olive oils of the commercial category virgin (VOO). Most of the collected oils belonged to the virgin category because this kind of oil presents a wider acidity range, thus a higher variability in the smoke point was expected. As stated in the Supplementary Materials (Table S1), the samples came from different Spanish regions and from different cultivars.

Each oil was homogenized, and sample aliquots were placed in vials of different volumes according to each determination. The vials were flushed with a nitrogen stream, sealed, and stored at −20 °C. Prior to analysis, the samples were thawed at room temperature.

### 2.2. Analytical Methods

All determinations described in this section were carried out in duplicate, except for the extinction coefficients, which were determined in triplicate.

#### 2.2.1. Trade Quality Indices

Determinations of acidity (FFA, expressed as % of oleic acid), peroxide value (PV, mEq O_2_/kg) and extinction coefficients (K_232_ and K_268_) were carried out according to the analytical methods described in the Commission Implementing Regulation (EU) 2022/2105 and its subsequent amendments [26].

#### 2.2.2. Moisture and Volatile Matter

The moisture and volatile matter content (MVM, in %) was determined following the vacuum oven method described in the AOCS Official Method Ca 2d-25 [30].

#### 2.2.3. Oxidative Stability

Oxidative stability was measured as the Oxidative Stability Index (OSI, in hours) according to the AOCS Official Method Cd 12b-92 [31], under the following conditions: temperature of 120 °C, an air flow rate of 20 L/h, using an 892 Professional Rancimat (Metrohm, Herisau, Switzerland).

#### 2.2.4. Fatty Acid Composition

A double methylation was used to prepare the FA methyl esters, which were then determined by gas chromatography-flame ionization detection (GC-FID), as described by Varona et al. [32]. The FA methyl esters were quantified by peak area normalization (peak area percentage).

#### 2.2.5. Lipophilic and Polar Phenolic Compounds

Tocopherols were determined according to the AOCS Official Method Ce 8-89 [33] with some modifications. The sample (1.5 g) was diluted with hexane in a 10 mL volumetric flask. Then, 20 mL of the solution were injected into an Agilent 1100 series HPLC (Agilent Technologies, Santa Clara, CA, USA) coupled to a Hewlett-Packard 1046A fluorescent detector. Separation was performed using a 4 × 3.0 mm precolumn (Phenomenex Security Guard Cartridge Silica) and a Luna silica column (150 × 4.6 mm i.d., 3 µm particle size and 100 Å pore size) from Phenomenex (Torrance, CA, USA). Elution was carried out isocratically using a mobile phase of hexane/1,4-dioxane (95/5, *v*/*v*). Detection was performed using an excitation wavelength of 290 nm and an emission wavelength of 320 nm. The results were expressed in mg/kg. Only α-tocopherol (α-T) was quantified, since the other tocopherol forms were either not detectable or present only in trace amounts.

Polar phenolic compounds were extracted as described by Vichi et al. [34] and analyzed by Ultra-High-Performance Liquid Chromatography coupled to a Diode Array Detector (UHPLC-DAD), adapting the chromatographic conditions of the IOC method COI/T.29/Doc No 29 [35] to a UHPLC system, as described by Nenadis et al. [36]. Briefly, 15 mL of the phenolic extract were injected into an Acquity-UPLC (Waters, Milford, MA, USA) coupled to a 2996 DAD (Waters, Milford, MA, USA). Separation was carried out using a Halo C18 Fused-Core column (100 × 2.1 mm and particle size of 2.7 µm) from Advanced Materials Technology (Wilmington, DE, USA). Elution was performed at a 0.4 mL/min flow rate and 30 °C, using as mobile phase ultrapure water (Milli-Q Millipore Corporation, Billerica, MA, USA)/formic acid (98:2, *v*/*v*) (solvent A), and methanol/acetonitrile (50:50, *v*/*v*) (solvent B). The solvent gradient changed as follows: from 96% (A)–4% (B), to 20% (B) at 5 min, to 45% (B) at 28 min, to 100% (B) at 30 min, 5 min maintenance until 35 min, then to 96% (A)–4% (B) at 36 min, followed by 5 min of equilibration. Detection was performed simultaneously at 335 nm and 280 nm. Identification was carried out according to the IOC method COI/T.29/Doc No 29 [35] and to Mateos et al. [37], and confirmed by high resolution mass spectrometry, using a Q-Exactive hybrid Orbitrap (Thermo Fisher Scientific, Bremen, Germany), under the described chromatographic conditions. Ion source and spectrometer conditions were as described by Vichi et al. [34]. The dialdehyde form of *p*-(hydroxyphenyl)ethanol elenolic acid aglycone (*p*-HPEA-EA) and one of the oxidized aldehydic and hydroxylic forms of oleuropein aglycone (3,4-(dihydroxyphenyl)ethanol elenolic acid or 3,4-DHPEA-EA) were not quantified by UHPLC-DAD analysis due to coelution.

Quantification was made using *o*-coumaric as the internal standard (IS) for flavones and *p*-hydroxyphenylacetic acid as the IS for the rest of the phenolic polar compounds, applying the response factors reported by Mateos et al. [37]. The results were expressed in mg/kg.

#### 2.2.6. Smoke Point

**Figure 1:**
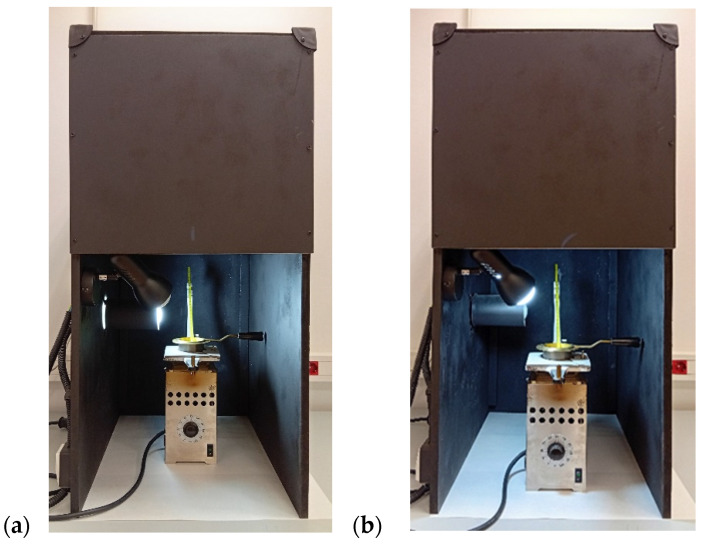
Apparatus used for smoke point determination: (**a**) lighting from the position relative to the sample specified by the AOCS Official Method Cc 9a-48 [1], and (**b**) lighting from the position specified by the Chinese National Standard GB/T 20795-2006 method [37].

The determination of the smoke point was carried out according to the AOCS Official Method Cc 9a-48 [1]. A cabinet was constructed following the dimensions specified in the AOCS method (Figure 1). A Cleveland apparatus (Humboldt Mfg. Co., Elgin, IL, USA), consisting of a Cleveland open flash cup, a heating plate, a cup support and an electric heater with rheostat control, was used. The Cleveland cup was filled with the oil test sample up to the filling line. The oil was heated rapidly for approximately 6 min to within 42 °C of its expected smoke point. The heating rate was then adjusted to achieve a temperature increase of 5–6 °C per minute, and the temperature was recorded when the test portion began to emit a thin continuous smoke.

After several repeatability tests with different samples conducted by two observers, it was concluded that the smoke point could be observed more clearly and precisely when using the light source position relative to the sample employed in the ST123 Oil Smoke Tester [38], in accordance with the Chinese National Standard GB/T 20795-2006 method [39], rather than the position recommended by the AOCS method.

In order to determine the most appropriate color temperature of the lighting source, three LED bulbs equivalent to a 100 W incandescent bulb, each emitting a luminous flux of 1521 lumens and at three different color temperatures (2700, 4000, and 6500 K), were used to measure the smoke point on the same oil (*n* = 4), and the coefficient of variation (CV) for each color temperature was calculated. The 2700 K bulb, which would be the equivalent to the color temperature recommended by the AOCS method, showed a CV three times higher (1.5%) than the 4000 K (0.4%) and 6500 K (0.4%) bulbs (see Table S2 in Supplementary Materials). Subsequently, the 4000 and 6500 K bulbs were tested again with a different oil (*n* = 6). The 6500 K LED bulb showed the lowest CV (0.4 vs. 0.8%) (Table S3, Supplementary Materials). Therefore, the measurement of the smoke point on the 48 olive oil samples was carried out with the 6500 K LED bulb.

### 2.3. Statistical Analysis

Partial least squares (PLS) regression was applied to the obtained data to explore the relationship between the smoke point and the other measured variables (FFA, PV, K_232_, K_268_, MVM, OSI, FA composition, α-T and polar phenolic compounds), using SIMCA v.13 (Sartorius AG, Göttingen, Germany). All the variables were standardized before the PLS analysis (mean centered and normalized to unit variance). The number of latent variables of each PLS model was selected according to the lowest root mean square error of cross-validation (RMSEcv, seven random cross-validation groups), and the maximum predictive power (Q^2^). According to Hotelling’s T^2^ and Q^2^ residual tests, no outliers were detected. Undesirable features, such as model overfitting, random behavior and low prediction power were assessed through permutation tests (with 20 permutations) and ANOVA (on the cross-validated residuals).

A Gaussian process is a stochastic process that defines a distribution over functions, fully determined by a mean and a covariance kernel [40]. Gaussian process regression (GPR) was applied to assess the relationship between the smoke point and the variables with the highest predictive value identified by the PLS models, while also considering the most commonly and readily measured parameters in virgin olive oils. The variables selected were FFAs, PV, K_232_, K_268,_ OSI, and saturated FA. Several kernels of varying smoothness—including smooth (squared exponential), moderately rough (Matérn 3/2 and 5/2), very rough (exponential, equivalent to Matérn 1/2), and scale-mixture (rational quadratic)—were evaluated, both in their isotropic and Automatic Relevance Determination (ARD) variants. These kernels are widely used in regression problems involving limited datasets and heterogeneous correlations, such as those considered in this study. To ensure comparability and reproducibility, all kernels were fitted within the same framework (MATLAB R2024a GPR implementation, MathWorks Inc., Natick, MA, USA), using log-marginal likelihood maximization with automatic estimation of hyperparameters (length-scale and variance) and noise level. No manual tuning or subjective adjustment was introduced. After preliminary evaluation, the kernels selected for comparison of predictive performance were the ARD squared exponential and the Matérn 3/2. Given the limited dataset (*n* = 48), leave-one-out cross-validation was employed to ensure statistical independence of test points and to obtain a realistic estimate of the generalization error.

## 3. Results and Discussion

### 3.1. Models Using Partial Least Squares Regression

#### 3.1.1. Model Building and Variable Selection

The data obtained from the characterization of the 48 virgin olive oils were analyzed using the PLS regression in order to build a full model (M1) capable of exploring the relationships between all the measured variables (FFA, PV, K_232_, K_268_, MVM, OSI, FA composition, α-T and polar phenolic compounds) and the smoke point. The selection of the variables included in the study was based on the fact that smoke formation during oil heating depends on the amount of FFAs [2], primarily derived from the hydrolysis of triacylglycerols, and on other volatile compounds present in the oil. These low-molecular-weight volatiles originate from a variety of reactions that may occur in the olive fruit, during oil extraction, or throughout storage [41,42]. The two processes that most strongly contribute to changes in the smoke point of an oil are the hydrolysis of triacylglycerols and the formation of volatile compounds resulting from lipid oxidation [14,15,25]. Accordingly, the variables analyzed in this study were: FFA content, which indicates the extent of hydrolysis [43]; the FA composition, which determines the susceptibility of the substrate to oxidation [44]; the amount of the main antioxidants (α-T and polar phenolic compounds); the concentrations of primary oxidation products (PV and K_232_), which possess pro-oxidant activity [45]; secondary oxidation products (K_268_); the oxidative stability (OSI); and, finally, the MVM. Regarding the FA profile, to build the full model (M1), the individual FA were grouped in 3 variables: saturated (SFAs), monounsaturated (MUFAs) and polyunsaturated (PUFAs) fatty acids. Similarly, the individual polar phenolic compounds were grouped in different variables according to the antioxidant activity and the simplicity of the measurement: total polar phenolic compounds, *o*-diphenols, secoiridoids (SEC), and simple phenols (hydroxytyrosol (HTy) and tyrosol (Ty)). To avoid redundancy, separate models were created, each incorporating different variables related to polar phenolic compounds. Based on the explained variance of the smoke point (R^2^Y) in the different models, the variables HTy, Ty and SEC were selected to be included in the full model (M1, see Table S4 in Supplementary Materials). Moreover, in Supplementary Materials, Table S5 shows the data used in the PLS regression models for smoke point prediction, and Tables S6 and S7 present the complete FA and polar phenol profiles.

With the aim of obtaining a simple equation to predict the smoke point with the minimum of measurements, the number of variables of the M1 model was reduced, obtaining the predictive models M2 and M3 (as defined below in Section 3.1.3). The criterion used for variable selection was based on the significance of the regression coefficient (centered and scaled, CoeffCS) of the prediction vector with respect to the smoke point, which is defined by the uncertainty measures (confidence intervals and standard error of cross validation or SEcv) obtained from the jack-knife method, which is used to estimate variance and bias.

#### 3.1.2. Relationship Between the Smoke Point and Other Quality and Compositional Parameters of Virgin Olive Oils

**Figure 2:**
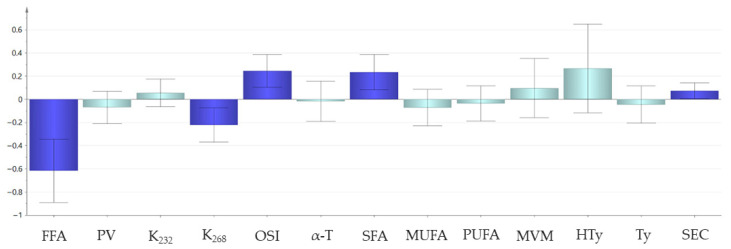
Regression coefficients (centered and scaled, CoeffCS) of the full PLS model (M1). The coefficients are significant (*p* < 0.05) when the confidence interval calculated from the jack-knife does not include the 0 (in dark blue). Abbreviations: FFAs, free fatty acids; PV, peroxide value; K_232_, extinction coefficient at 232 nm; K_268_, extinction coefficient at 268 nm; OSI, oxidative stability index; α-T, α-tocopherol; SFAs, saturated fatty acids; MUFA, monounsaturated fatty acids; PUFAs, polyunsaturated fatty acids; MVM, moisture and volatile matter; HTy, hydroxytyrosol; Ty, tyrosol; SEC, secoiridoids.

Figure 2 shows the regression coefficients (CoeffCS) of the variables introduced in the full PLS model (M1). These coefficients are useful to interpret the influence of the variables on the smoke point. In this model, FFAs, K_268_, OSI, SFAs and SEC were significantly related to the smoke point, as shown by the dark blue color.

FFA, which showed the highest regression coefficient, was negatively related to the smoke point, in agreement with previous literature [21,24]. In contrast, SFA was positively related to the smoke point. This parameter ranged from 12.5% to 19.8%, consisting mainly of palmitic acid and, to a lesser extent, stearic acid (see Table S6 in the Supplementary Materials). This positive relationship could be related to the higher oxidative stability of SFAs and, consequently, to their lower susceptibility to generate volatile oxidation compounds. Actually, the energy required to generate an alkyl radical increases in this order: linoleic (C18:2) < oleic (C18:1) < stearic acid (C18:0), which justifies their different oxidation rate during thermal oxidation or autoxidation [12]. Therefore, unsaturated FA oxidize and break down into volatile compounds more easily; consequently, oils with comparable FA chain lengths but higher unsaturation tend to have lower smoke points. This explains why virgin olive oils with higher SFA content exhibit higher smoke points.

The regression coefficients of PV, K_232_ and K_268_ showed that the smoke point was more closely related to the secondary oxidation products (K_268_, significantly correlated) than to the primary oxidation products (PV and K_232_, not significantly correlated). This is justified by the fact that some secondary oxidation compounds are volatile, therefore they contribute more directly than the primary oxidation compounds to the appearance of smoke.

Lastly, the positive and significant regression coefficients of OSI and SEC indicated that these two parameters contributed to increasing the smoke point. Assuming that smoke is partly composed of decomposition products from lipid oxidation, and considering the antiradical activity of phenolic antioxidants that slows down this process, the positive and significant relationship between the smoke point and the oxidative stability of the oil is coherent and consistent with previous studies [23]. The study by Mateos et al. [46] showed that α-T contributes to a lesser extent to the oxidative stability of virgin olive oil than *o*-diphenolic compounds (included in the SEC content), which could explain the lack of significance of its regression coefficient in the present model. In this context, it is worth noting that SEC are present in considerably higher concentrations in virgin olive oils compared to refined and seed oils [47]. However, their content progressively undergoes hydrolysis and oxidation during storage [48] and thermal processing [49], leading to a gradual decrease in their concentration and, consequently, in their effectiveness in delaying the formation of volatile oxidation products.

#### 3.1.3. Predictive Models Using PLS

With the aim of obtaining a simple predictive model, an intermediate model with the significant variables of the full model M1 was built (FFA, K_268_, OSI, SFA and SEC), in which SEC presented a non-significant regression coefficient. Excluding SEC, the predictive model M2 was built, which included FFAs, K_268_, OSI and SFAs. The 4 variables introduced in the model were significant, with FFAs presenting the highest regression coefficient (Table 1).

For a predictive model to be easily applicable, it is essential to incorporate the fewest possible variables. Therefore, new models fixing FFAs and reducing one variable at a time were built (models M2 *, M2 ** and M2 ***, Table 1). Table 2 shows the main features of all models. All simplified models showed lower prediction power than M2, as shown by the higher prediction errors (RMSEcv) and the lower Q^2^, which represents the fraction of the smoke point variance that can be predicted by the model as estimated through cross-validation. Decreases in the prediction power when reducing variables are usual, but when seeking a simple predictive model based on the fewest possible analytical determinations, it is advisable to find the best compromise between the model’s predictive power and the number of variables used.

Among the models obtained by omitting one variable (M2 *, M2 **, and M2 ***, Table 1), the best performance was achieved by M2 ** (highest Q^2^ and lowest RMSEcv, Table 2), which included FFAs, SFAs, and OSI. This suggests that K_268_ had a lower influence on the prediction of the smoke point of virgin olive oils. The samples used in this study belonged to the EVOO and VOO commercial categories, and all of them had K_268_ values within the limits established by EU regulation [26] (Table S5, Supplementary Materials). Consequently, the oils exhibited low levels of secondary oxidation, which may explain why K_268_ contributed less to the smoke point prediction compared with other variables. The models M3 * and M3 were built from model M2 **, omitting OSI in M3 and SFAs in M3 *. While omitting OSI (model M3) led to almost similar Q^2^ and RMSEcv value to M2 **, the exclusion of SFAs from the model (model M3 *) entailed a decrease in the model’s prediction power (Table 2). In this way, a simple predictive model (M3) was obtained using only two analytical determinations, being FFAs and SFAs, as the best predictors of the smoke point of virgin olive oils.

**Figure 3:**
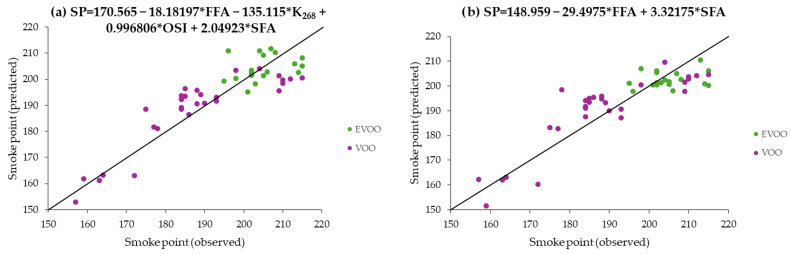
Predicted versus observed smoke point values for each sample (*n* = 48), based on the PLS predictive models M2 (**a**) and M3 (**b**). The smoke prediction equation is shown at the top of each graph. In each graph, the bisecting line (y = x) is shown. Abbreviations: SP, smoke point; FFA, free fatty acids; K_268_, extinction coefficient at 268 nm; OSI, oxidative stability index; SFA, saturated fatty acids; EVOO, extra virgin olive oil; VOO, virgin extra olive oil.

The graphs displaying the smoke point predicted values (*Y*-axis) against the values observed (*X*-axis) by the models obtained before (M2) and after variable reduction (M3), along with their equations, are shown in Figure 3. A low dispersion was observed in both models around the bisecting lines, as expected from the obtained RMSEcv values. Overall, even if the predictive power decreased when reducing variables, the increase in the RMSEcv for the smoke point from M1 (based on 13 variables) to M3 (based on FFAs and SFAs) was only of 0.59 °C. Using this model, a coefficient of determination (R^2^) of 0.77 was achieved (from results represented in Figure 3b), a result comparable to that of a previously reported smoke point prediction model for refined edible oils, which was based on NIR assessment and required specific instrumentation [29].

### 3.2. Models Using Gaussian Process Regression

Gaussian process regression (GPR) with different kernels was also employed to evaluate the relationship between the smoke point and the variables with the highest predictive value identified by the PLS models, while also incorporating the most readily measurable and commonly assessed parameters in virgin olive oil. The selected variables were FFAs, PV, K_232_, K_268_, OSI, and SFAs. Prior to assessing the relationship between smoke point and the selected variables, an extensive kernel search was conducted. All commonly used Gaussian process kernels of varying smoothness were evaluated, including smooth kernels (squared exponential, ARD squared exponential, and linear + ARD squared exponential residuals), moderately rough kernels (Matérn 3/2, ARD Matérn 3/2, Matérn 5/2, ARD Matérn 5/2, and linear + Matérn 3/2 residuals), very rough kernels (exponential and ARD exponential), and scale-mixture kernels (rational quadratic and ARD rational quadratic), complemented with a pure linear kernel. These kernels were assessed to determine their predictive accuracy of the smoke point using only the variables included in model M3 (FFAs and SFAs). As stated in the Supplementary Materials (Table S8), the mean absolute deviations (MAE) across all nonlinear kernels ranged narrowly between 2.9 °C and 4.2 °C and mean relative errors (MRE) between 1.45% and 2.14%. All nonlinear kernels, regardless of smoothness, outperformed both the pure linear kernel and the classical linear regression (model M3). This confirms that introducing a nonlinear covariance structure captures the weakly nonlinear dependence between smoke point and compositional parameters, even though the differences among specific kernels fall within approximately ±0.5 °C, a range that lies below the experimental uncertainty of smoke point measurements. Thus, the ARD squared exponential and the Matérn 3/2 kernels were selected as standard, representative, and complementary forms of functional smoothness. The squared exponential kernel assumes infinitely differentiable relationships and therefore represents the most restrictive smooth prior, suitable for highly regular processes. In contrast, the Matérn 3/2 kernel corresponds to once-differentiable sample paths, capturing moderately rough yet continuous behavior. By using both, we effectively span the two limiting cases commonly considered in Gaussian process regression, thereby enabling a direct examination of how the inferred dependence between the smoke point and compositional parameters behaves under both very smooth and moderately rough prior assumptions. Table 3 shows the errors of Gaussian models based on the ARD squared exponential kernel and on the Matérn 3/2 kernel for the selected variables. It can be observed that the mean errors (MAE and MRE) are consistently lower for the Gaussian models based on the Matérn 3/2 kernel, which indicates that they predicted slightly better.

Table 3 also shows that the Gaussian predictive models (G4_SE_, G5_SE_, G4_M3/2_, and G5_M3/2_) yielded much lower prediction errors than the PLS models selected in the previous section (M2 and M3). In fact, the Gaussian models that use only the FFA variable (G1_SE_ and G1_M3/2_) perform better than either of the two PLS models (M2 and M3), which use four and two variables, respectively.

**Figure 4:**
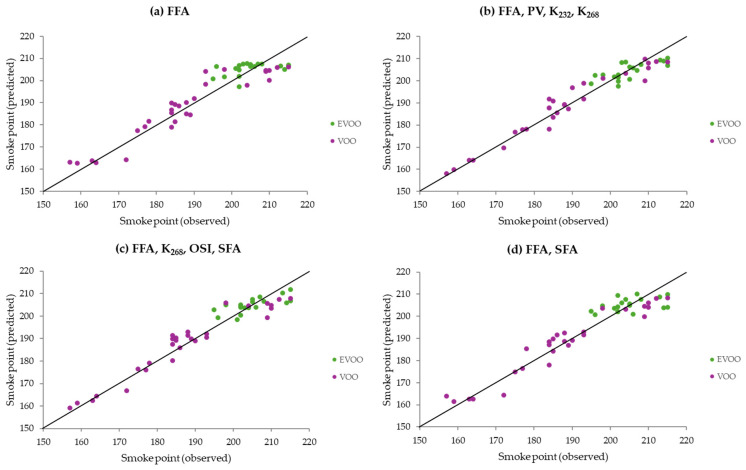
Predicted versus observed smoke point values for each sample (*n* = 48), based on the Gaussian predictive models G1_M3/2_ (**a**), G3_M3/2_ (**b**), G4_M3/2_ (**c**), and G5_M3/2_ (**d**). The predictor variables used in each model are indicated at the top of each graph. In each graph, the bisecting line (y = x) is shown. Abbreviations: FFAs, free fatty acids; PV, peroxide value; K_232_, extinction coefficient at 232 nm; K_268_, extinction coefficient at 268 nm; OSI, oxidative stability index; SFAs, saturated fatty acids; EVOO, extra virgin olive oil; VOO, virgin olive oil.

When comparing the scatter around the bisecting lines in Figure 3 (models M2 and M3) and Figure 4 (models G1_M3/2_, G3_M3/2_, G4M_3/2_, and G5_M3/2_), it becomes clear that the Gaussian models based on the Matérn 3/2 kernel outperformed the PLS models. There was a closer agreement between the predicted and observed smoke points of the samples for the Gaussian models.

From Table 3 and Figure 3 and Figure 4, it can be seen that model G1_M3/2_, which used only the FFA variable, predicted the smoke point of virgin olive oils very well. The coefficient of determination (R^2^) in this case was 0.88 (from results represented in Figure 4a), a value higher than that of a previously reported model for refined edible oils which required the use of the NIR technique [29]. This is particularly relevant since FFA is a commonly and readily determined quality parameter. In addition, model G3_M3/2_, which used four commonly and readily determined quality parameters (FFA, PV, K_232_, K_268_) in virgin olive oils, was the best predictive model.

## 4. Conclusions

The AOCS procedure for smoke point determination was reassessed and optimized. It was found that the light source position recommended by the Chinese National Standard method provided clearer and more precise observations than that of the AOCS method. Furthermore, testing different light color temperatures revealed that the 6500 K LED bulb significantly reduced variability in the measurements. Therefore, all smoke point determinations were carried out using this setup, ensuring higher accuracy and repeatability.

This study deepened the understanding of the smoke point of virgin olive oil and its relationship with key quality and compositional parameters, showing that the smoke point strongly depends on its FFA content, a routinely measured quality parameter in virgin olive oils, which is negatively correlated with it. SFAs and OSI showed positive correlations, while secondary oxidation products (K_268_) also had a negative correlation, confirming that the smoke point reflects the oil’s oxidative and hydrolytic state.

A simple PLS regression model (M3) using only FFAs and SFAs predicted the smoke point with good accuracy, but Gaussian models performed better. In particular, Gaussian models based on the Matérn 3/2 kernel yielded consistently lower mean errors than those based on the ARD squared exponential kernel. Remarkably, even the simplest Gaussian models using only FFAs as a predictor variable outperformed the PLS models that relied on multiple variables. This is especially relevant because Gaussian models based solely on FFAs allow accurate prediction of the smoke point, potentially eliminating the need for direct smoke point testing, which is not routinely performed and requires a highly trained observer for precise and accurate measurement. In addition, among all models, G3_M3/2_, which combines four commonly and readily determined quality parameters in virgin olive oils (FFAs, PV, K_232_, and K_268_), achieved the best overall predictive performance. These predictive models (G1_M3/2_, G2_M3/2_, G3_M3/2_) based on combinations of FFAs, PV, K_232_, and K_268,_ could be very useful for the olive oil sector since they are based on easily determined parameters that are routinely analyzed by oil producers, depending on their analytical capacity.

These findings not only advance the understanding of the relationship between smoke point and oil composition but also provide an accurate and precise method for smoke point determination and several potential predictive models for assessing the oil’s suitability for deep-frying. While these models represent a significant advancement in identifying the variables with the greatest predictive power as well as the first application of Gaussian process regression to smoke point prediction, they have only been internally validated, and further external and interlaboratory validation will be necessary in future studies to confirm the generalizability of the model. Nevertheless, these predictive models can be updated and enhanced in the future by adding new information obtained from samples of subsequent olive oil harvests, collected from different laboratories, thereby increasing their robustness.

## Figures and Tables

**Table 1:** Regression coefficients centered and scaled (CoeffCS) and jack-knife standard errors of cross-validation (SEcv) of the reduced models created from the full PLS model M1.

	M2		M2 *		M2 **		M2 ***		M3 *		M3	
	CoeffCS	SEcv	CoeffCS	SEcv	CoeffCS	SEcv	CoeffCS	SEcv	CoeffCS	SEcv	CoeffCS	SEcv
FFA	**−0.498** ^1^	0.115	**−0.66**	0.184	**−0.674**	0.192	**−0.736**	0.15	**−0.751**	0.109	**−0.807**	0.161
K_268_	**−0.26**	0.157	−0.108	0.262			−0.133	0.263				
OSI	**0.341**	0.084	0.231	0.264	**0.216**	0.13			**0.163**	0.147		
SFA	**0.18**	0.14			**0.309**	0.256	**0.315**	0.251			**0.291**	0.145

^1^ Coefficients in bold type are significant (absolute value greater than SEcv). Abbreviations: *, **, ***, intermediate models; FFAs, free fatty acids; K_268_, extinction coefficient at 268 nm; OSI, oxidative stability index; SFAs, saturated fatty acids.

**Table 2:** Main features of the PLS models.

Model	Variables	Latent Variables	R^2^X (%)	R^2^Y (%)	Q^2^ (%)	RMSEcv (°C)
M1	13	3	65.1	85.9	78.6	7.09
M2	4	1	42.7	78.3	76.9	7.38
M2 *	3	2	70.6	73.5	70.3	8.34
M2 **	3	2	74.3	78.5	74.8	7.72
M2 ***	3	2	73.8	77.2	72.8	7.92
M3 *	2	2	100	72.5	71.6	8.18
M3	2	1	52.2	76.7	75	7.68

*n* = 48. Abbreviations: *, **, ***, intermediate models; R^2^X, amount of variation in the X-block used by the model; R^2^Y, amount of variation in the Y-block (smoke point) explained by the model; Q^2^, determination coefficient; RMSEcv, root mean square error of cross-validation. Variables used in each model are stated in Figure 2 (M1) and Table 1 (rest of models).

**Table 3:** Errors of the selected Gaussian and PLS predictive models.

Model	Variables	MAE (°C)	MRE (%)	Max AE (°C)	Max RE (%)
G1_SE_	FFA	4.69	2.40	**10.82**	**5.61**
G2_SE_	FFA, PV	4.69	2.40	10.82	5.61
G3_SE_	FFA, PV, K_232_, K_268_	3.40	1.72	9.41	4.80
G4_SE_	FFA, K_268_, OSI, SFA	3.57	1.82	**9.71**	**4.51**
G5_SE_	FFA, SFA	3.87	1.99	**9.62**	**4.82**
G1_M3/2_	FFA	**4.50**	**2.29**	11.20	5.80
G2_M3/2_	FFA, PV	**3.65**	**1.84**	**10.50**	**4.90**
G3_M3/2_	FFA, PV, K_232_, K_268_	**3.09**	**1.56**	**9.08**	**4.34**
G4_M3/2_	FFA, K_268_, OSI, SFA	**3.42**	**1.74**	9.77	4.68
G5_M3/2_	FFA, SFA	**3.84**	**1.96**	10.96	5.10
M2	FFA, K_268_, OSI, SFA	5.74	2.93	14.96	7.69
M3	FFA, SFA	6.17	3.19	20.55	11.55

*n* = 48; In order to compare the Gaussian models, the lowest errors are in bold type. Abbreviations: MAE, mean absolute error; MRE, mean relative error; Max AE, maximum absolute error; Max RE, maximum relative error; GX_SE,_, Gaussian models based on the ARD squared exponential kernel; GX_M3/2_, Gaussian models based on the Matérn 3/2 kernel; M2 and M3, PLS selected models; FFAs, free fatty acids; PV, peroxide value, K_232_, extinction coefficient at 232 nm; K_268_, extinction coefficient at 268 nm; OSI, oxidative stability index; SFAs, saturated fatty acids.

## Data Availability

The datasets generated for this study are available on request to the corresponding author due to privacy.

## Supplementary Materials

The following supporting information can be downloaded at: https://www.mdpi.com/article/10.3390/foods14234099/s1, Table S1: Commercial category, campaign, cultivar and origin of the oils used in the study; Table S2: Smoke point (°C) measurements performed on the same oil using three lightbulbs with three different color temperatures: 2700, 4000, and 6500 K (*n* = 4); Table S3: Smoke point (°C) measurements performed on the same oil using two lightbulbs with two different color temperatures: 4000 K and 6500 K (*n* = 6); Table S4: Main characteristics of the models developed with samples of virgin oils combining different variables related to the polyphenol profile for their selection to build the predictive model M1; Table S5: Compositional data matrix used for developing the smoke point predictive models; Table S6: Fatty acid profile (%) of the olive oil samples; Table S7: Polar phenolic compounds profile (mg/kg) of the olive oil samples; Table S8: Evaluation of the predictive accuracy of the smoke point using the variables free fatty acids (FFA) and saturated fatty acids (SFA), and different Gaussian process regression kernels.

## References

[1] AOCS (2017). Smoke, Flash and Fire Points Cleveland Open Cup Method; Official Method Cc 9a-48. Official Methods and Recommended Practices of the American Oil Chemists’ Society.

[2] Tsaknis J., Spiliotis V., Lalas S., Gergis V., Dourtoglou V. (1999). Quality Changes of Moringa Oleifera, Variety Mbololo of Kenya, Seed Oil during Frying. Grasas Aceites.

[3] Dobarganes C., Márquez-Ruiz G. (2013). Analysis of Used Frying Oils. Lipid Technol..

[4] Katragadda H.R., Fullana A., Sidhu S., Carbonell-Barrachina Á.A. (2010). Emissions of Volatile Aldehydes from Heated Cooking Oils. Food Chem..

[5] Abdullahi K.L., Delgado-Saborit J.M., Harrison R.M. (2013). Emissions and Indoor Concentrations of Particulate Matter and Its Specific Chemical Components from Cooking: A Review. Atmos. Environ..

[6] Ganesan K., Sukalingam K., Xu B. (2019). Impact of Consumption of Repeatedly Heated Cooking Oils on the Incidence of Various Cancers: A Critical Review. Crit. Rev. Food Sci. Nutr..

[7] Ma S., Liu W., Meng C., Dong J., Zhang S. (2023). Temperature-dependent particle mass emission rate during heating of edible oils and their regression models. Environ. Pollut..

[8] Ölzigen S. (2020). Cooking as a Chemical Reaction: Culinary Science with Experiments.

[9] Culinary Institute of America (2002). The Professional Chef.

[10] Rossell J.B., Rossell J.B. (2001). Factors Affecting the Quality of Frying Oils and Fats. Frying: Improving Quality.

[11] Dobarganes M.C., Velasco J., Márquez-Ruiz G. (2002). La Calidad de Los Aceites y Grasas de Fritura. Aliment. Nutr. Salud.

[12] Choe E., Min D.B. (2007). Chemistry of Deep-Fat Frying Oils. J. Food Sci..

[13] Österreichisches Lebensmittelbuch IV (2019). Auflage Codexkapitel/B 30/Speisefette, Speiseöle, Streichfette Und Andere Fetterzeugnisse.

[14] Matthäus B. (2006). Utilization of High-Oleic Rapeseed Oil for Deep-Fat Frying of French Fries Compared to Other Commonly Used Edible Oils. Eur. J. Lipid Sci. Technol..

[15] Fan H.Y., Sharifudin M.S., Hasmadi M., Chew H.M. (2013). Frying Stability of Rice Bran Oil and Palm Olein. Int. Food Res. J..

[16] Quaglia G.B., Bucarelli F.M., Rossell J.B. (2001). Effective Process Control in Frying. Frying: Improving Quality.

[17] Kalogianni E.P., Georgiou D., Romaidi M., Exarhopoulos S., Petridis D., Karastogiannidou C., Dimitreli G., Karakosta P. (2017). Rapid Methods for Frying Oil Quality Determination: Evaluation with Respect to Legislation Criteria. J. Am. Oil Chem. Soc..

[18] Mallikarjunan P.K., Ngadi M.O., Chinnan M.S. (2010). Breaded Fried Foods.

[19] Bockisch M. (1998). Fats and Oils Handbook.

[20] De Alzaa F., Guillaume C., Ravetti L. (2018). Evaluation of Chemical and Physical Changes in Different Commercial Oils during Heating. Acta Sci. Nutr. Health.

[21] Matthäus B., Brühl L. (2014). Quality Parameters for the Evaluation of Cold-Pressed Edible Argan Oil. J. Für Verbraucherschutz Und Leb..

[22] Gunstone F.D. (2011). Vegetable Oils in Food Technology: Composition, Properties and Uses.

[23] Yen G.-C., Shao C.-H., Chen C.-J., Duh P.-D. (1997). Effects of Antioxidant and Cholesterol on Smoke Point of Oils. LWT-Food Sci. Technol..

[24] Wu Y.T., Fang B., Wu H.Y., Shen Y.M. (2016). Establishment of Mathematical Relationships between Smoke Point and Minor Compounds in Vegetable Oils Using Principal Component Regression Analysis. Chem. Eng. Trans..

[25] Al-Dabbas M.M., Al-Jaloudi R., Abdullah M.A., Abughoush M. (2023). Characterization of olive oil volatile compounds after elution through selected bleaching materials—Gas chromatography–mass spectrometry analysis. Molecules.

[26] Commission Implementing Regulation (EU) (2022). 2022/2105 of 29 July 2022 Laying down Rules on Conformity Checks of Marketing Standards for Olive Oil and Methods of Analysis of the Characteristics of Olive Oil. Off. J. Eur. Union.

[27] Li X., Bremer G.C., Connell K.N., Ngai C., Anh Q., Pham T., Wang S., Flynn M., Ravetti L., Guillaume C. (2016). Changes in Chemical Compositions of Olive Oil under Different Heating Temperatures Similar to Home Cooking. J. Food Chem. Nutr..

[28] Lam H.Y., Roy P.K., Chattopadhyay S. (2020). Thermal Degradation in Edible Oils by Surface Enhanced Raman Spectroscopy Calibrated with Iodine Values. Vib. Spectrosc..

[29] Öğütcü M., Aydeniz B., Büyükcan M.B., Yılmaz E. (2012). Determining Frying Oil Degradation by Near-Infrared Spectroscopy Using Chemometric Techniques. J. Am. Oil Chem. Soc..

[30] AOCS (2017). Moisture and Volatile Matter, Vacuum Oven Method; Official Method Ca 2d-25. Official Methods and Recommended Practices of the American Oil Chemists’ Society.

[31] AOCS (2017). Oil Stability Index (OSI); Official Method Cd 12b-92. Official Methods and Recommended Practices of the American Oil Chemists’ Society.

[32] Varona E., Tres A., Rafecas M., Vichi S., Barroeta A.C., Guardiola F. (2021). Methods to Determine the Quality of Acid Oils and Fatty Acid Distillates Used in Animal Feeding. MethodsX.

[33] AOCS (2017). Determination of Tocopherols and Tocotrienols in Vegetable Oils and Fats by HPLC; Official Method Ce 8-89. Official Methods and Recommended Practices of the American Oil Chemists’ Society.

[34] Vichi S., Cortés-Francisco N., Caixach J. (2013). Insight into Virgin Olive Oil Secoiridoids Characterization by High-Resolution Mass Spectrometry and Accurate Mass Measurements. J. Chromatogr. A.

[35] IOC (2009). Determination of Biophenols in Olive Oils by HPLC.

[36] Nenadis N., Mastralexi A., Tsimidou M.Z., Vichi S., Quintanilla-Casas B., Donarski J., Bailey-Horne V., Butinar B., Miklavčič M., García González D.L. (2018). Toward a Harmonized and Standardized Protocol for the Determination of Total Hydroxytyrosol and Tyrosol Content in Virgin Olive Oil (VOO). Extraction Solvent. Eur. J. Lipid Sci. Technol..

[37] Mateos R., Espartero J.L., Trujillo M., Ríos J.J., León-Camacho M., Alcudia F., Cert A. (2001). Determination of Phenols, Flavones, and Lignans in Virgin Olive Oils by Solid-Phase Extraction and High-Performance Liquid Chromatography with Diode Array Ultraviolet Detection. J. Agric. Food Chem..

[38] ST123 Oil Smoke Tester-Edible Oil Tester Oil Smoke Point Analyzer for Animal and Vegetable Oils. https://gonoava.en.made-in-china.com/product/utqYnPBHsiVz/China-Edible-Oil-Tester-Oil-Smoke-Point-Analyzer-for-Animal-and-Vegetable-Oils.html?pv_id=1j1r14a56bd4&faw_id=1j1r14bdd4ec&bv_id=1j1r14dnsd19.

[39] (2006). Determination of Smoking Point for Vegetable Fats and Oils.

[40] Rasmussen C.E., Williams C.K.I. (2006). Gaussian Processes for Machine.

[41] Aparicio R., Harwood J. (2013). Handbook of Olive Oil: Analysis and Properties.

[42] Kalua C.M., Allen M.S., Bedgood D.R., Bishop A.G., Prenzler P.D., Robards K. (2007). Olive oil volatile compounds, flavour development and quality: A critical review. Food Chem..

[43] Di Pietro M.E., Mannu A., Mele A. (2020). NMR Determination of Free Fatty Acids in Vegetable Oils. Processes.

[44] Morelló J.-R., Motilva M.-J., Tovar M.-J., Romero M.-P. (2004). Changes in commercial virgin olive oil (cv Arbequina) during storage, with special emphasis on the phenolic fraction. Food Chem..

[45] Kim H.J., Hahm T.S., Min D.B. (2007). Hydroperoxide as a prooxidant in the oxidative stability of soybean oil. J. Am. Oil Chem. Soc..

[46] Mateos R., Domínguez M.M., Espartero J.L., Cert A. (2003). Antioxidant Effect of Phenolic Compounds, α-Tocopherol, and Other Minor Components in Virgin Olive Oil. J. Agric. Food Chem..

[47] Owen R.W., Mier W., Giacosa A., Hull W.E., Spiegelhalder B., Bartsch H. (2000). Phenolic Compounds and Squalene in Olive Oils: The Concentration and Antioxidant Potential of Total Phenols, Simple Phenols, Secoiridoids, Lignans and Squalene. Food Chem. Toxicol..

[48] Díez-Betriu A., Romero A., Ninot A., Tres A., Vichi S., Guardiola F. (2023). Subzero Temperature Storage to Preserve the Quality Attributes of Veiled Virgin Olive Oil. Foods.

[49] Klisović D., Novoselić A., Lukić M., Kraljić K., Brkić Bubola K. (2024). Thermal-Induced Alterations in Phenolic and Volatile Profiles of Monovarietal Extra Virgin Olive Oils. Foods.

