# Supplementary material for: Deciphering the Complexity of Smoke Point in Virgin Olive Oils to Develop Simple Predictive Models
Source: Foods. 2025 Nov 28;14(23):4099. doi: 10.3390/foods14234099 (PMC12692607; doi:10.3390/foods14234099)
Supplement: Supplementary file 1 [file foods-14-04099-s001.zip › foods-3950458-supplementary.pdf]

Supplementary Materials

Deciphering the Complexity of Smoke Point in Virgin Olive Oils to Develop Simple Predictive Models

Anna Díez-Betriu, Beatriz Quintanilla-Casas, Josep J. Masdemont, Alba Tres, Stefania Vichi * and Francesc Guardiola

* Correspondence: stefaniavichi@ub.edu

Table S1. Commercial category, campaign, cultivar and origin of the oils used in the study.

Sample	Category	Campaign	Cultivar	Origin
1	EVOO	2017/18	Picual	Andalusia
2	EVOO	2017/18	Arbequina	Andalusia
3	EVOO	2017/18	Picual	Catalonia
4	EVOO	2017/18	Arbequina	Catalonia
5	EVOO	2017/18	Hojiblanca	Catalonia
6	EVOO	2017/18	Picual	Andalusia
7	EVOO	2017/18	Arbequina	Catalonia
8	VOO	2017/18	Coupage	Catalonia
9	VOO	2017/18	Coupage	Catalonia
10	VOO	2017/18	Coupage	Catalonia
11	VOO	2017/18	Coupage	Catalonia
12	VOO	2017/18	Coupage	Catalonia
13	EVOO	2017/18	Hojiblanca	Andalusia
14	EVOO	2017/18	Hojiblanca	Andalusia
15	EVOO	2017/18	Hojiblanca	Valencia
16	VOO	2017/18	Coupage	Catalonia
17	VOO	2017/18	Coupage	Catalonia
18	EVOO	2017/18	Hojiblanca	Andalusia
19	EVOO	2017/18	Picual	Andalusia
20	EVOO	2017/18	Arbequina	Catalonia
21	EVOO	2017/18	Picual	Andalusia

22	VOO	2017/18	Coupage	Catalonia
23	VOO	2017/18	Coupage	Catalonia
24	VOO	2017/18	Coupage	Catalonia
25	VOO	2017/18	Coupage	Catalonia
26	VOO	2017/18	Coupage	Catalonia
27	VOO	2017/18	Coupage	Catalonia
28	EVOO	2017/18	Arbequina	Catalonia
29	EVOO	2018/19	Arbequina	Catalonia
30	EVOO	2018/19	Picual	Catalonia
31	VOO	2018/19	Coupage	Catalonia
32	EVOO	2018/19	Arbequina	Andalusia
33	EVOO	2018/19	Picual	Andalusia
34	VOO	2018/19	Coupage	Catalonia
35	VOO	2018/19	Coupage	Catalonia
36	VOO	2018/19	Coupage	Catalonia
37	VOO	2018/19	Coupage	Catalonia
38	VOO	2018/19	Coupage	Catalonia
39	VOO	2018/19	Coupage	Catalonia
40	VOO	2018/19	Coupage	Catalonia
41	VOO	2018/19	Coupage	Catalonia
42	VOO	2018/19	Coupage	Catalonia
43	VOO	2018/19	Coupage	Catalonia
44	VOO	2018/19	Coupage	Catalonia
45	VOO	2018/19	Coupage	Catalonia
46	VOO	2018/19	Coupage	Catalonia
47	VOO	2018/19	Coupage	Catalonia
48	VOO	2018/19	Coupage	Catalonia

Abbreviations: EVOO, extra virgin olive oil; VOO, virgin olive oil.

Table S2. Smoke point (ºC) measurements performed on the same oil using three lightbulbs with three different color temperatures: 2700, 4000, and 6500 K (n = 4).

	2700 K	4000 K	6500 K
1	232	222	222
2	224	222	224
3	226	224	224
4	228	222	224
Mean	227.5	222.5	223.5
Standard deviation	3.4	1	1
Coefficient of variation (%)	1.5	0.4	0.4

Table S3. Smoke point (ºC) measurements performed on the same oil using two lightbulbs with two different color temperatures: 4000 K and 6500 K (n = 6).

	4000 K	6500 K
1	200	204
2	202	202
3	204	202
4	202	202
5	204	202
6	204	202
Mean	202.7	202.3
Standard deviation	1.6	0.8
Coefficient of variation (%)	0.8	0.4

Table S4. Main characteristics of the models developed with samples of virgin oils combining different variables related to the polyphenol profile for their selection to build the predictive model M1.

Variables assessed	n	no. variables	Latent variables	R^2X	R^2Y	Q^2	RMSEcv
Total polyphenols	48	11	3	66	83.2	78	7.22
o-diphenols	48	11	3	65.5	83.6	78.8	7.07
HTy + Ty, SEC	48	12	3	66	84.2	77.1	7.3
**HTy, Ty, SEC**	**48**	**13**	**3**	**65.1**	**85.9**	**78.6**	**7.09**

Abbreviations: R^2X, amount of variation in the X-block used by the model; R^2Y, amount of variation in the Y-block (smoke point) explained by the model; Q^2, determination coefficient; RMSEcv, root mean square error of cross-validation; HTy, hydroxytyrosol, Ty, tyrosol, SEC, secoiridoids. Variables selected for the M1 model are in bold.

Table S5. Compositional data matrix used for developing the smoke point predictive models.

Sample	Category	Smoke point (°C)	FFA (% oleic acid)	PV (mEqO$_2$/kg)	K_{232}	K_{268}	OSI (h)	α-T (mg/kg)	MVM (% weight)	SFA (%)	MUFA (%)	PUFA (%)	HTy (mg/kg)	Ty (mg/kg)	SEC (mg/kg)
1	EVOO	208	0.067	2.0	1.36	0.11	21.4	336.2	0.036	16.74	78.68	4.57	1.17	2.61	405.24
2	EVOO	213	0.087	1.8	1.51	0.12	13.6	446.7	0.065	19.29	67.15	13.56	1.01	2.04	431.18
3	EVOO	204	0.062	2.5	1.43	0.12	23.6	348.6	0.098	16.63	78.79	4.58	2.18	1.87	250.30
4	EVOO	215	0.076	3.3	1.60	0.11	17.1	331.4	0.103	17.91	72.05	10.04	0.98	1.65	394.30
5	EVOO	205	0.073	3.9	1.45	0.11	13.8	331.5	0.086	16.19	74.83	8.98	1.09	2.91	228.44
6	EVOO	207	0.068	4.5	1.40	0.11	21.3	408.6	0.083	17.50	77.45	5.05	1.87	2.47	257.99
7	EVOO	202	0.177	5.9	1.57	0.09	8.6	264.8	0.088	18.80	69.55	11.65	2.44	2.48	120.06
8	VOO	185	0.423	4.1	1.41	0.08	8.2	275.4	0.053	17.65	69.65	12.70	1.14	2.31	233.54
9	VOO	188	0.310	5.9	1.62	0.10	10.4	264.0	0.059	16.52	73.41	10.07	7.89	5.56	211.86
10	VOO	209	0.117	8.7	1.93	0.10	8.3	222.3	0.061	15.74	75.34	8.92	15.18	10.61	59.15
11	VOO	172	1.573	15.5	2.03	0.15	5.6	259.5	0.097	17.38	73.63	8.99	14.32	12.55	46.52
12	VOO	190	0.220	7.4	1.68	0.09	7.0	333.9	0.046	14.28	73.36	12.35	2.26	4.83	82.17
13	EVOO	202	0.138	4.4	1.60	0.15	19.5	378.8	0.060	16.72	74.72	8.56	6.23	5.68	457.16
14	EVOO	206	0.090	3.8	1.48	0.11	16.9	336.3	0.056	15.56	76.28	8.15	2.98	3.40	344.39
15	EVOO	203	0.069	6.6	1.66	0.09	7.6	316.7	0.041	16.36	74.43	9.21	0.61	2.55	62.62
16	VOO	189	0.318	6.3	1.52	0.09	8.3	179.8	0.038	16.17	73.55	10.29	0.80	3.46	155.16
17	VOO	184	0.513	7.1	1.51	0.09	6.8	190.6	0.043	16.20	72.44	11.36	0.72	4.43	118.66
18	EVOO	201	0.101	4.7	1.70	0.15	13.0	444.9	0.059	16.39	75.36	8.24	2.71	3.99	226.95
19	EVOO	205	0.090	4.2	1.29	0.09	18.3	484.9	0.069	16.69	78.79	4.52	1.59	4.58	166.92

Sample	Category	Smoke point (°C)	FFA (% oleic acid)	PV (mEqO$_2$/kg)	K_{232}	K_{268}	OSI (h)	α-T (mg/kg)	MVM (% weight)	SFA (%)	MUFA (%)	PUFA (%)	HTy (mg/kg)	Ty (mg/kg)	SEC (mg/kg)
20	EVOO	215	0.087	5.7	1.61	0.11	17.7	240.4	0.089	16.21	74.46	9.32	7.28	4.83	271.61
21	EVOO	202	0.109	7.3	1.81	0.13	18.1	379.9	0.093	16.71	78.33	4.97	7.41	6.73	142.40
22	VOO	209	0.113	5.2	1.45	0.09	10.5	283.4	0.100	16.86	73.27	9.87	1.06	1.51	89.13
23	VOO	210	0.112	9.9	1.76	0.09	8.0	210.2	0.026	17.21	72.18	10.61	6.12	3.34	77.02
24	VOO	212	0.096	8.8	1.73	0.10	8.9	212.7	0.026	17.51	71.77	10.72	8.62	4.24	71.51
25	VOO	215	0.093	8.9	1.71	0.10	9.1	205.1	0.029	17.59	71.70	10.71	9.51	5.12	70.36
26	VOO	210	0.155	8.5	1.82	0.10	7.6	196.5	0.034	17.88	70.15	11.98	8.52	6.14	66.38
27	VOO	204	0.173	8.4	1.78	0.10	9.6	157.5	0.042	19.82	66.67	13.52	2.35	4.49	130.61
28	EVOO	214	0.107	6.2	1.78	0.09	12.1	206.6	0.065	16.56	73.71	9.73	1.52	2.13	140.81
29	EVOO	198	0.141	5.6	1.62	0.11	8.8	377.2	0.052	18.72	69.31	11.97	0.63	1.83	70.38
30	EVOO	202	0.082	5.0	1.48	0.15	16.3	404.9	0.033	17.74	76.30	5.96	1.22	1.73	106.96
31	VOO	178	0.411	8.8	2.15	0.19	5.6	220.3	0.032	18.58	68.76	12.66	2.93	6.60	71.47
32	EVOO	195	0.149	5.0	1.50	0.09	8.8	309.8	0.035	17.01	71.79	11.20	1.04	3.05	96.01
33	EVOO	196	0.090	5.6	1.47	0.10	23.7	387.9	0.039	15.53	80.53	3.94	2.27	4.41	165.27
34	VOO	164	1.383	12.0	1.81	0.16	5.5	243.9	0.034	16.52	70.86	12.62	9.06	8.79	62.90
35	VOO	157	1.499	27.5	2.81	0.22	3.9	102.6	0.080	17.28	73.24	9.48	13.32	14.72	51.62
36	VOO	184	0.298	7.3	1.75	0.10	8.8	241.7	0.036	16.26	74.22	9.52	3.54	5.83	103.25
37	VOO	159	1.716	6.4	1.45	0.12	5.9	273.1	0.062	15.98	74.89	9.13	0.74	1.89	85.34
38	VOO	175	0.569	5.9	1.37	0.08	7.5	239.5	0.028	15.37	75.66	8.97	0.52	2.49	112.76
39	VOO	198	0.107	4.2	1.44	0.09	13.3	339.4	0.047	16.47	73.90	9.63	5.26	3.16	135.31

Sample	Category	Smoke point (°C)	FFA (% oleic acid)	PV (mEqO$_2$/kg)	K_{232}	K_{268}	OSI (h)	α-T (mg/kg)	MVM (% weight)	SFA (%)	MUFA (%)	PUFA (%)	HTy (mg/kg)	Ty (mg/kg)	SEC (mg/kg)
40	VOO	163	1.268	12.7	2.25	0.15	2.7	188.4	0.041	15.21	72.54	12.25	7.42	9.89	50.65
41	VOO	177	0.507	8.4	1.98	0.12	6.3	240.7	0.053	14.70	73.82	11.48	9.94	5.60	100.41
42	VOO	188	0.236	10.4	1.75	0.13	8.5	230.2	0.058	16.22	74.28	9.49	3.87	3.14	57.98
43	VOO	185	0.245	5.6	1.47	0.08	6.3	184.7	0.045	15.56	75.46	8.98	0.66	1.92	36.50
44	VOO	186	0.251	8.8	1.75	0.17	10.2	271.4	0.049	16.22	74.20	9.57	5.69	3.38	80.09
45	VOO	193	0.116	5.8	1.43	0.09	11.0	293.2	0.043	12.52	78.58	8.90	1.66	1.26	64.95
46	VOO	184	0.237	8.0	1.58	0.14	14.6	292.8	0.043	14.78	79.76	5.46	3.94	3.84	173.60
47	VOO	184	0.276	7.4	1.57	0.12	7.8	217.7	0.047	15.34	75.87	8.79	8.39	13.11	59.16
48	VOO	193	0.169	8.3	1.48	0.14	14.2	217.9	0.040	14.05	80.83	5.12	4.79	4.50	195.98

Abbreviations: FFA, free fatty acids; PV, peroxide value; K_{232}, extinction coefficient at 232 nm; K_{268}, extinction coefficient at 268 nm OSI, oxidative stability index; α-T, α-tocopherol; MVM, moisture and volatile matter; SFA, saturated fatty acids; MUFA, monounsaturated fatty acids; PUFA, polyunsaturated fatty acids; HTy, hydroxytyrosol; Ty, tyrosol; SEC, secoiridoids; EVOO, extra virgin olive oil; VOO, virgin olive oil.

Table S6. Fatty acid profile (%) of the olive oil samples.

Sample	Category	C14:0	C15:0	C16:0	C16:1n9	C16:1n7	C17:0	C17:1	C18:0	C18:1n9	C18:1n7	C18:2n6	C20:0	C18:3n3	C20:1n9	C21:0	C22:0	C23:0	C24:0
1	EVOO	0.01	0.01	13.27	0.08	1.21	0.05	0.08	2.73	74.63	2.43	3.81	0.42	0.76	0.26	0.02	0.13	0.03	0.08
2	EVOO	0.02	0.01	16.33	0.15	1.52	0.15	0.30	2.04	61.73	3.14	12.86	0.45	0.70	0.31	0.02	0.15	0.03	0.08
3	EVOO	0.01	0.01	13.16	0.08	0.92	0.08	0.12	2.71	75.23	2.17	3.77	0.42	0.81	0.27	0.01	0.12	0.03	0.07
4	EVOO	0.02	0.01	14.74	0.16	1.08	0.20	0.35	2.28	67.46	2.70	9.37	0.43	0.66	0.30	0.02	0.13	0.02	0.07
5	EVOO	0.02	0.01	12.73	0.12	0.75	0.17	0.28	2.59	71.36	2.01	8.06	0.43	0.92	0.33	0.01	0.13	0.03	0.07
6	EVOO	0.02	0.01	14.18	0.08	1.30	0.04	0.07	2.61	73.22	2.52	4.24	0.39	0.82	0.25	0.01	0.12	0.03	0.08
7	EVOO	0.02	0.01	15.78	0.15	1.55	0.15	0.28	2.20	64.32	2.97	11.05	0.41	0.60	0.29	0.02	0.13	0.03	0.07
8	VOO	0.02	0.01	14.47	0.14	1.25	0.08	0.15	2.42	65.04	2.78	11.99	0.43	0.70	0.28	0.02	0.13	0.03	0.06
9	VOO	0.02	0.01	13.19	0.15	1.24	0.10	0.18	2.50	68.89	2.64	9.38	0.45	0.69	0.31	0.02	0.15	0.03	0.07
10	VOO	0.02	0.01	12.76	0.16	0.93	0.15	0.26	2.14	71.19	2.48	8.35	0.43	0.57	0.32	0.02	0.14	0.02	0.06
11	VOO	0.02	0.01	14.14	0.13	1.31	0.15	0.30	2.42	68.92	2.68	8.17	0.40	0.82	0.28	0.02	0.12	0.03	0.07
12	VOO	0.01	0.01	10.56	0.14	0.58	0.11	0.15	2.85	70.40	1.73	11.69	0.47	0.67	0.36	0.02	0.16	0.03	0.07
13	EVOO	0.02	0.01	12.52	0.12	0.94	0.18	0.27	3.27	71.24	1.86	7.71	0.47	0.85	0.28	0.02	0.13	0.03	0.07
14	EVOO	0.01	0.01	11.64	0.12	0.79	0.15	0.24	3.09	73.05	1.80	7.36	0.43	0.79	0.28	0.02	0.12	0.03	0.06
15	EVOO	0.02	0.01	13.48	0.14	1.15	0.12	0.24	2.08	69.91	2.66	8.43	0.41	0.78	0.33	0.02	0.13	0.03	0.07
16	VOO	0.01	0.00	12.42	0.13	0.92	0.06	0.11	2.92	69.99	2.11	9.63	0.49	0.65	0.29	0.02	0.15	0.02	0.07
17	VOO	0.01	0.00	12.46	0.14	0.92	0.06	0.10	2.93	68.85	2.14	10.67	0.47	0.69	0.29	0.02	0.15	0.02	0.06
18	EVOO	0.02	0.02	12.32	0.12	0.83	0.20	0.30	3.07	72.05	1.76	7.26	0.48	0.98	0.30	0.02	0.15	0.04	0.09
19	EVOO	0.01	0.01	13.51	0.09	1.29	0.05	0.10	2.46	74.49	2.56	3.73	0.39	0.79	0.26	0.02	0.13	0.03	0.08
20	EVOO	0.01	0.01	13.32	0.14	1.04	0.12	0.23	2.14	70.31	2.45	8.81	0.40	0.51	0.29	0.02	0.12	0.02	0.05

Sample	Category	C14:0	C15:0	C16:0	C16:1n9	C16:1n7	C17:0	C17:1	C18:0	C18:1n9	C18:1n7	C18:2n6	C20:0	C18:3n3	C20:1n9	C21:0	C22:0	C23:0	C24:0
21	EVOO	0.01	0.01	13.18	0.09	1.16	0.05	0.09	2.81	74.39	2.34	4.24	0.41	0.72	0.25	0.02	0.12	0.03	0.07
22	VOO	0.02	0.01	13.48	0.17	0.94	0.18	0.28	2.49	69.41	2.18	9.24	0.45	0.63	0.29	0.02	0.14	0.02	0.06
23	VOO	0.02	0.00	14.05	0.18	1.36	0.18	0.00	2.31	67.48	2.88	9.98	0.43	0.63	0.29	0.02	0.13	0.02	0.06
24	VOO	0.02	0.00	14.35	0.18	1.42	0.18	0.00	2.29	66.93	2.96	10.07	0.43	0.64	0.29	0.02	0.13	0.02	0.06
25	VOO	0.02	0.00	14.46	0.18	1.44	0.18	0.00	2.27	66.80	3.00	10.07	0.43	0.64	0.29	0.02	0.13	0.02	0.06
26	VOO	0.02	0.00	15.03	0.16	1.52	0.13	0.00	2.03	64.97	3.20	11.38	0.42	0.59	0.29	0.02	0.14	0.02	0.06
27	VOO	0.02	0.00	16.99	0.14	1.71	0.14	0.00	2.02	61.13	3.42	12.96	0.42	0.55	0.28	0.02	0.13	0.02	0.06
28	EVOO	0.02	0.00	13.54	0.16	1.22	0.16	0.00	2.20	69.31	2.73	9.18	0.41	0.55	0.29	0.02	0.13	0.02	0.06
29	EVOO	0.02	0.01	15.79	0.16	1.41	0.14	0.28	2.06	64.54	2.61	11.23	0.43	0.74	0.32	0.02	0.14	0.03	0.07
30	EVOO	0.01	0.01	13.77	0.09	0.93	0.11	0.17	3.08	73.24	1.58	5.01	0.47	0.95	0.30	0.02	0.14	0.05	0.08
31	VOO	0.02	0.01	15.76	0.17	1.96	0.12	0.26	2.00	63.04	3.01	11.94	0.41	0.72	0.33	0.02	0.13	0.04	0.07
32	EVOO	0.02	0.01	14.18	0.16	1.31	0.11	0.23	2.06	67.24	2.55	10.55	0.40	0.65	0.30	0.02	0.13	0.03	0.07
33	EVOO	0.01	0.01	12.02	0.09	0.95	0.05	0.09	2.83	77.28	1.85	3.15	0.39	0.79	0.26	0.01	0.11	0.04	0.07
34	VOO	0.02	0.01	12.91	0.12	0.77	0.15	0.24	2.68	67.77	1.63	11.85	0.47	0.77	0.34	0.02	0.15	0.03	0.08
35	VOO	0.02	0.01	14.05	0.14	1.30	0.13	0.26	2.42	68.75	2.48	8.80	0.42	0.68	0.30	0.02	0.13	0.03	0.07
36	VOO	0.01	0.01	13.25	0.15	1.07	0.13	0.24	2.22	70.26	2.18	8.88	0.42	0.64	0.32	0.02	0.13	0.03	0.06
37	VOO	0.01	0.01	12.33	0.14	0.92	0.07	0.13	2.82	71.35	2.03	8.43	0.48	0.70	0.31	0.02	0.15	0.03	0.07
38	VOO	0.01	0.00	11.89	0.14	0.80	0.06	0.11	2.70	72.48	1.81	8.32	0.46	0.65	0.31	0.02	0.14	0.03	0.06
39	VOO	0.02	0.01	13.72	0.16	1.18	0.12	0.24	1.95	69.58	2.40	9.02	0.41	0.61	0.33	0.02	0.14	0.03	0.07
40	VOO	0.01	0.01	11.11	0.14	0.58	0.10	0.14	3.18	70.09	1.23	11.55	0.52	0.71	0.36	0.02	0.16	0.03	0.07
41	VOO	0.01	0.01	10.65	0.14	0.55	0.09	0.13	3.14	71.45	1.19	10.79	0.52	0.69	0.36	0.02	0.16	0.03	0.07

Sample	Category	C14:0	C15:0	C16:0	C16:1n9	C16:1n7	C17:0	C17:1	C18:0	C18:1n9	C18:1n7	C18:2n6	C20:0	C18:3n3	C20:1n9	C21:0	C22:0	C23:0	C24:0
42	VOO	0.02	0.01	13.29	0.16	1.04	0.14	0.26	2.13	70.40	2.12	8.95	0.41	0.54	0.31	0.02	0.14	0.02	0.06
43	VOO	0.01	0.00	12.70	0.17	0.96	0.13	0.24	2.09	71.77	2.01	8.44	0.40	0.54	0.31	0.02	0.13	0.02	0.06
44	VOO	0.02	0.01	13.27	0.15	1.02	0.13	0.25	2.12	70.41	2.06	9.02	0.42	0.55	0.32	0.02	0.15	0.02	0.07
45	VOO	0.01	0.01	7.71	0.19	0.36	0.18	0.27	3.92	76.61	0.77	8.13	0.47	0.77	0.38	0.02	0.13	0.03	0.05
46	VOO	0.01	0.00	10.11	0.13	0.66	0.06	0.11	3.95	77.34	1.27	4.76	0.43	0.71	0.26	0.02	0.12	0.03	0.05
47	VOO	0.01	0.01	11.75	0.16	0.97	0.13	0.23	2.76	72.45	1.73	8.05	0.44	0.73	0.33	0.02	0.14	0.03	0.06
48	VOO	0.01	0.00	9.93	0.12	0.52	0.07	0.12	3.45	78.64	1.18	4.51	0.39	0.62	0.26	0.01	0.11	0.02	0.05

Abbreviations: EVOO, extra virgin olive oil; VOO, virgin olive oil.

Table S7. Polar phenolic compounds profile (mg/kg) of the olive oil samples.

Sample	Category	1	2	3	4	5	6	7	8	9	10	11	12	13	14	15	16	17	18
1	EVOO	1.17	2.61	56.48	57.24	68.96	65.92	70.20	86.43	0.04	0.04	0.00	0.00	0.42	2.52	6.29	2.53	3.68	1.75
2	EVOO	1.01	2.04	268.03	2.90	3.75	10.86	120.68	24.96	0.00	0.00	0.00	0.00	1.11	0.00	1.48	1.82	4.50	1.95
3	EVOO	2.18	1.87	69.52	27.01	31.98	26.57	55.61	39.61	0.02	0.01	0.04	0.00	0.15	1.51	2.89	3.00	1.83	0.92
4	EVOO	0.98	1.65	264.05	5.66	7.38	8.24	81.16	27.80	0.00	0.00	0.00	0.00	0.29	0.90	1.10	2.56	1.66	0.35
5	EVOO	1.09	2.91	96.15	8.00	9.86	11.92	76.13	26.37	0.06	0.00	0.00	0.01	0.25	0.93	1.75	1.94	3.30	2.25
6	EVOO	1.87	2.47	57.52	21.90	26.63	28.37	57.12	66.45	0.00	0.03	0.00	0.00	0.26	2.27	3.57	2.41	2.11	1.09
7	EVOO	2.44	2.48	55.84	1.42	0.91	4.49	47.33	10.06	0.06	0.06	0.00	0.02	0.27	0.29	2.79	2.37	3.56	0.89
8	VOO	1.14	2.31	84.41	2.22	3.33	4.90	121.59	17.10	0.00	0.00	0.00	0.00	0.17	0.49	1.29	1.59	3.41	1.16
9	VOO	7.89	5.56	81.00	2.92	4.40	9.44	94.72	19.38	0.02	0.31	0.00	0.10	0.46	0.90	2.43	2.35	3.37	1.24
10	VOO	15.18	10.61	18.62	0.89	0.55	3.37	27.47	8.25	0.05	1.21	0.00	1.72	0.34	0.97	2.00	2.36	4.02	1.56
11	VOO	14.32	12.55	10.07	0.74	6.17	1.75	18.09	9.70	0.12	0.72	0.00	0.46	0.34	1.74	2.77	2.34	1.10	0.43
12	VOO	2.26	4.83	15.69	2.65	3.76	7.43	27.05	25.60	0.09	0.01	0.01	0.01	0.22	1.37	4.65	1.66	1.13	0.57
13	EVOO	6.23	5.68	133.94	23.51	29.81	43.77	140.11	86.01	0.00	0.27	0.00	0.18	0.27	0.00	24.78	1.43	4.55	1.71
14	EVOO	2.98	3.40	96.67	30.54	40.78	38.83	76.68	60.87	0.00	0.11	0.00	0.05	0.13	0.00	4.29	1.23	5.03	2.21
15	EVOO	0.61	2.55	10.32	3.31	3.97	11.66	23.49	9.87	0.40	0.04	0.00	0.14	0.33	0.00	2.69	2.23	6.14	3.06
16	VOO	0.80	3.46	46.60	2.18	3.82	8.88	82.51	11.17	0.03	0.01	0.00	0.00	0.05	0.00	0.93	1.71	1.39	0.95
17	VOO	0.72	4.43	42.88	1.09	2.11	2.55	62.21	7.82	0.02	0.00	0.00	0.00	0.06	0.00	1.24	0.99	1.16	0.70
18	EVOO	2.71	3.99	58.76	12.57	16.03	31.91	69.62	38.04	0.00	0.26	0.00	0.34	0.33	0.00	2.95	0.98	4.73	2.71
19	EVOO	1.59	4.58	34.44	3.74	6.23	13.44	53.21	55.87	0.02	0.06	0.00	0.00	0.50	0.00	3.35	3.21	1.95	1.74
20	EVOO	7.28	4.83	186.56	1.23	2.58	3.41	57.42	20.42	0.00	0.36	0.00	0.10	0.28	0.00	1.06	2.49	4.29	0.81

Sample	Category	1	2	3	4	5	6	7	8	9	10	11	12	13	14	15	16	17	18
21	EVOO	7.41	6.73	29.89	7.94	11.55	14.96	42.11	35.96	0.15	3.64	0.06	2.87	1.60	0.00	2.20	2.51	2.41	1.15
22	VOO	1.06	1.51	40.34	0.68	0.63	2.57	35.51	9.41	0.05	0.24	0.00	0.09	0.44	0.00	0.68	2.29	2.24	0.52
23	VOO	6.12	3.34	33.21	0.42	0.57	2.18	34.99	5.66	0.01	1.13	0.00	0.78	0.39	0.00	1.32	2.05	3.43	0.64
24	VOO	8.62	4.24	32.12	0.54	0.81	2.50	29.09	6.47	0.01	1.42	0.00	0.77	0.48	0.00	1.55	2.16	3.68	0.66
25	VOO	9.51	5.12	31.12	0.57	0.80	2.52	28.82	6.53	0.01	1.47	0.00	0.79	0.50	0.00	1.57	2.17	3.75	0.67
26	VOO	8.52	6.14	31.82	0.92	0.59	3.16	24.07	5.82	0.06	0.97	0.00	0.42	0.59	0.00	2.04	2.27	4.85	0.97
27	VOO	2.35	4.49	78.96	1.25	0.58	4.71	33.97	11.14	0.04	1.40	0.00	0.38	0.92	0.00	0.83	2.64	2.85	0.80
28	EVOO	1.52	2.13	84.06	0.83	0.90	2.18	42.62	10.21	0.00	0.89	0.00	0.32	0.26	0.00	1.38	2.66	3.97	0.78
29	EVOO	0.63	1.83	32.04	1.25	0.93	4.36	23.65	8.15	0.09	0.01	0.00	0.00	0.59	0.00	0.68	2.42	1.27	0.38
30	EVOO	1.22	1.73	24.02	8.58	11.75	15.47	28.18	18.97	0.06	0.04	0.00	0.00	0.21	0.00	1.36	2.50	1.26	0.51
31	VOO	2.93	6.60	18.83	1.15	0.66	8.15	36.42	6.27	0.13	0.33	0.00	0.92	0.55	0.00	1.38	1.53	1.81	0.73
32	EVOO	1.04	3.05	34.48	1.46	0.77	8.86	39.39	11.05	0.29	0.01	0.00	0.03	0.51	0.00	1.47	2.01	2.39	0.96
33	EVOO	2.27	4.41	45.87	8.42	12.17	14.23	44.85	39.72	0.06	0.07	0.00	0.02	0.41	0.00	4.12	2.94	2.36	1.11
34	VOO	9.06	8.79	12.39	1.14	3.76	4.88	21.07	19.66	0.27	0.23	0.00	0.17	0.12	0.00	1.66	2.90	1.76	0.76
35	VOO	13.32	14.72	16.46	0.50	2.76	2.34	23.17	6.39	0.41	4.27	0.00	2.27	0.28	0.00	0.92	2.00	1.24	0.40
36	VOO	3.54	5.83	42.17	0.75	1.40	4.36	44.33	10.23	0.02	0.37	0.00	0.32	0.21	0.00	1.55	1.84	2.65	0.99
37	VOO	0.74	1.89	25.34	0.86	1.67	1.82	48.99	6.66	0.04	0.11	0.00	0.00	0.05	0.00	0.63	1.33	1.24	0.65
38	VOO	0.52	2.49	26.95	0.88	0.81	1.84	75.60	6.68	0.03	0.00	0.00	0.00	0.06	0.00	0.47	1.33	1.13	0.81
39	VOO	5.26	3.16	76.81	2.01	1.10	9.01	36.46	9.92	0.14	0.76	0.00	0.10	0.73	0.00	1.85	2.15	2.06	1.69
40	VOO	7.42	9.89	7.88	2.51	4.66	5.45	17.13	13.03	0.18	0.59	0.03	0.69	0.30	0.00	1.11	1.52	0.97	0.33
41	VOO	9.94	5.60	28.43	7.01	10.62	8.97	25.48	19.89	0.09	0.97	0.00	0.62	0.50	0.00	1.30	1.38	102.47	30.55

Sample	Category	1	2	3	4	5	6	7	8	9	10	11	12	13	14	15	16	17	18
42	VOO	3.87	3.14	23.38	0.71	0.35	2.60	27.06	3.89	0.11	0.73	0.00	0.42	0.10	0.00	1.08	1.75	2.99	0.96
43	VOO	0.66	1.92	1.58	0.50	0.29	3.27	27.06	3.79	0.32	0.18	0.00	0.17	0.03	0.00	1.02	1.60	2.75	1.08
44	VOO	5.69	3.38	41.19	1.03	0.49	2.28	31.09	4.01	0.03	0.62	0.00	0.27	0.18	0.00	0.96	1.92	2.86	0.91
45	VOO	1.66	1.26	22.81	3.90	5.69	5.56	13.09	13.90	0.00	0.25	0.05	0.06	0.05	0.00	3.70	0.37	1.04	0.45
46	VOO	3.94	3.84	47.56	5.55	8.27	14.50	44.12	53.58	0.00	0.44	0.00	0.34	0.09	0.00	4.38	1.16	0.82	0.27
47	VOO	8.39	13.11	13.54	2.47	3.72	7.87	19.29	12.29	0.10	0.30	0.00	0.30	0.27	0.00	1.71	1.20	2.38	1.21
48	VOO	4.79	4.50	55.73	7.99	11.58	20.59	39.35	60.74	0.00	0.66	0.00	0.37	0.12	0.00	2.73	1.21	0.78	0.30

1. Hydroxytyrosol
2. Tyrosol
3. 3,4-DHPEA-EDA
4. Oleuropein
5. Dialdehydic form of 3,4 -DHPEA-EA
6. Dialdehydic and hydroxylic forms of 3,4-HPEA-EA aglycon
7. *p*-HPEA-EDA
8. Aldehydic and hydroxylic forms of *p*-HPEA-EDA

9 and 10. Oxidized form of 3,4-DHPEA-EDA
11 and 12. Oxidized form of *p*-HPEA-EDA
13. Oxidized form of the aldehydic and hydroxylic forms of 3,4-HPEA-EA
14. Oxidized form of the aldehydic and hydroxylic forms of *p*-HPEA-EA
15. Tyrosol acetate
16. Pinoresinol + 1-acetoxypinoresinol
17. Luteolin
18. Apigenin

Abbreviations: EVOO, extra virgin olive oil; VOO, virgin olive oil.

Table S8. Evaluation of the predictive accuracy of the smoke point using the variables free fatty acids (FFA) and saturated fatty acids (SFA), and different Gaussian process regression kernels.

GPR Kernels	MAE (°C)	MRE (%)	Max AE (°C)	Max RE (%)
Squared exponential	4.16	2.14	11.03	5.13
**ARD squared exponential**	**3.87**	**1.99**	**9.62**	**4.82**
Linear + ARD squared exponential residuals	3.50	1.79	9.74	4.61
**Matérn 3/2**	**3.84**	**1.96**	**10.96**	**5.10**
ARD Matérn 3/2	3.74	1.91	9.55	4.46
Linear + Matérn 3/2 residuals	3.65	1.87	10.47	5.00
Matérn 5/2	4.02	2.06	11.04	5.14
ARD Matérn 5/2	3.81	1.95	9.57	4.65
Exponential (Matérn 1/2)	2.86	1.45	9.03	4.22
ARD Exponential	3.18	1.62	8.73	4.06
Rational Quadratic	4.14	2.13	11.02	5.12
ARD Rational Quadratic	3.83	1.96	9.58	4.66
Linear (pure)	6.15	3.18	19.52	10.97

Abbreviations: GPR, Gaussian process regression; MAE, mean absolute error; MRE, mean relative error; Max AE, maximum absolute error; Max RE, maximum relative error; ARD, automatic relevance determination. The models included in the paper are the ones in bold type.
